# Practice patterns and approach to kidney biopsy in lupus: a collaboration of the Midwest pediatric nephrology consortium and the childhood arthritis and rheumatology research alliance

**DOI:** 10.1186/s12969-015-0024-x

**Published:** 2015-06-19

**Authors:** Scott E. Wenderfer, Jerome C. Lane, Ibrahim F. Shatat, Emily von Scheven, Natasha M. Ruth

**Affiliations:** Pediatric Nephrology, Texas Children’s Hospital, Baylor College of Medicine, Houston, TX USA; Pediatric Nephrology, Ann and Robert H. Lurie Children’s Hospital of Chicago, Feinberg School of Medicine, Northwestern University, Chicago, IL USA; Pediatric Nephrology, Medical University of South Carolina Children’s Hospital, Charleston, SC USA; Pediatric Rheumatology, University of California San Francisco, San Francisco, CA USA; Pediatric Rheumatology, Medical University of South Carolina Children’s Hospital, Charleston, SC USA

**Keywords:** SLE, Nephritis, Rheumatology, Renal, Nephrology, Pediatrics, Survey, CARRA, MWPNC

## Abstract

**Background:**

There is no clear consensus regarding optimal indications or timing of initial or repeat kidney biopsy in the management of pediatric systemic lupus erythematosus (pSLE).

**Methods:**

A web-based survey was designed to assess current practice patterns among pediatric nephrologists and pediatric rheumatologists and distributed to members of Midwest Pediatric Nephrology Consortium (MWPNC) and Childhood Arthritis and Rheumatology Research Alliance (CARRA).

**Results:**

Respondents included 111 rheumatologists and 71 nephrologists from 65 and 34 centers, respectively. Numbers of years in sub-specialty practice were comparable. Rheumatologists and nephrologists frequently collaborate in the care of children with lupus nephritis (LN). More than 90 % of respondents refer patients to each either other after diagnosing LN. Over 60 % describe shared decision making regarding when to perform kidney biopsy and how to interpret biopsy findings. Many pediatric nephrologists consider biopsy to be of higher risk for complication in pSLE and alter their standard pre-or post-biopsy management.

**Conclusions:**

It is uncommon for pediatric nephrologists to manage LN without input from pediatric rheumatologists and *vice versa*. Consensus exists between specialties in general, and practice differences that exist occur between individual physicians rather than between specialties. A systematic approach to biopsy may result in improved health related outcomes in pSLE.

**Electronic supplementary material:**

The online version of this article (doi:10.1186/s12969-015-0024-x) contains supplementary material, which is available to authorized users.

## Background

Approximately 50 % of patients with pediatric-onset systemic lupus erythematosus (pSLE) will develop some form of lupus nephritis (LN) [1]. This results in increased risk for renal failure, cardiovascular disease and death. LN is a serious disease that requires prolonged therapy with complex treatment plans and often toxic therapy. The histopathology seen on kidney biopsy is often central to the choice of therapy and disease management. The American College of Rheumatology (ACR) developed guidelines to assist physicians caring for adult patients with SLE in deciding when to perform a first kidney biopsy (Table 1) [2]. Several pediatric rheumatologists and nephrologists worry that applying these guidelines to all children may be inappropriate, and the editorial accompanying the publication of the ACR guidelines warns that they should not be applied to children or even adult males [3]. However, pediatric specific guidelines are currently not available.

**Table 1:** American College of Rheumatology biopsy guidelines (adapted from Table 2 in reference 2)

1)	Increasing serum creatinine without compelling alternative cause^a^, or
2)	Confirmed proteinuria of ≥ 1 g per 24-hrs^b^, or
3)	Combinations of the following, assuming the findings are confirmed within a short period of time and in the absence of alternative causes:
•	Proteinuria ≥ 0.5 g per 24-hrs^b^ plus hematuria^c^, or
•	Proteinuria ≥ 0.5 g per 24-hrs^b^plus cellular casts.

^a^Alternative causes include sepsis, hypovolemia, or medication

^b^For proteinuria, either timed urine collections or spot urine protein/creatinine ratios are acceptable, but cutoffs for the latter are not specified

^c^Hematuria is defined as ≥ 5 RBCs per high powered field

Although classification of LN according to renal histopathology is essential in guiding appropriate therapy, there remains disagreement over the classification system that best predicts prognosis or response to therapy [4]. The World Health Organization (WHO) classification is still used by some pathologists even after publication of a newer classification by the International Society of Nephrology in collaboration with the Renal Pathology Society (ISN/RPS) in 2003 [5]. The activity index (AI) and chronicity index (CI) as developed by Morel-Marogel *et al.* and refined by Austin *et al.* [6] for proliferative classes of LN are now used for all classes by some pathologists. Three groups have published the utility of histopathological classification, AI, and CI in their pSLE cohorts in Canada [7, 8] and Thailand [9], but additional evidence from other pediatric centers is needed.

The Joint European League Against Rheumatism (EULAR) and the European Renal Association-European Dialysis and Transplant Association (ERA-EDTA) recommend repeat kidney biopsy 6-months after induction therapy for LN in children and adults [1], but this has not been universally adopted. The topic has been reviewed [4, 10, 11] and approaches for repeat biopsy have been proposed [12–14], but consensus is lacking. Practices include the assessment of decreases in AI or changes in class after induction therapy, increases in AI or CI in refractory disease, absence of disease activity prior to weaning maintenance medication, or confirmation of relapse in patients with kidney flares. Reports of findings from repeat biopsies performed 6–to 9-months after 3–, 6–, and 9–months of induction therapy for proliferative LN in pSLE have been published [15–17], but there have been no studies of repeat biopsy in children with class V membranous LN.

There is considerable variation in pre-biopsy preparation and biopsy technique utilized in children undergoing biopsy for LN [18, 19]. Pediatric nephrologists at many centers perform their own biopsies, but other centers rely on interventional radiology to perform all or select “high risk” biopsies [19]. SLE patients are often taking aspirin, non-steroidal anti-inflammatories, or anticoagulation which most but not all physicians recommend stopping before biopsy, but the variation in length of time between stopping and performance of biopsy has not been studied. The risk of complications after kidney biopsy in children is between 0–14 % [11, 18, 20–22], and risks of major complications such as prolonged gross hematuria and large perinephric hematoma range from 3–7 %. Routine U/S performed 2 weeks after kidney biopsy identified intra-renal or peri-renal hematomas in 40 % of children, all of which resolved without further complication [23]. Reported predictors of complications include anemia and azotemia, but concerns exist with platelet dysfunction, recent or current anti-coagulation, solitary kidney, small kidneys, and advanced CKD [24]. Although pSLE patients were included in these cohorts, the numbers were too small to assess the effects of underlying systemic vasculitis, anti-phospholipid antibodies, or hypertension on complication rates. Some centers transfuse for anemia and thrombocytopenia in order to biopsy prior to starting induction therapy, whereas other centers treat presumptively and biopsy weeks or months into treatment.

Routine post-biopsy care also varies tremendously among pediatric nephrologists [18, 19, 23, 25]. Some observe all patients overnight whereas others discharge low risk children home after same day procedure [23]. For pSLE, practice variation may in part reflect the involvement of different sub-specialists in the care of the patients, most importantly pediatric nephrologists and rheumatologists.

In this study, we describe practice patterns related to kidney biopsy of children with LN, compare the variations in practice among and between pediatric nephrologists and rheumatologists, and assess how these two sub-specialties interact in clinical decision making. We present and discuss the results of a survey addressing specifically in pSLE the approach to first biopsy, approach to repeat biopsy, mechanisms for obtaining biopsies, and methods for interpretation of results.

## Methods

Practice patterns were assessed with an online survey sent to 300 members of the CARRA and 160 members of the MWPNC via SurveyMonkey in January 2014. The survey included 26 questions designed to delineate current clinical practice in the approach to renal biopsy in pSLE. Respondents had the option of disclosing their institution. Respondents were uniquely identified by IP address and date of response. Nineteen questions were directed towards both pediatric rheumatologists and pediatric nephrologists, two specifically directed towards pediatric rheumatologists, and 5 specifically towards pediatric nephrologists (See Additional files 1 and 2). The survey questions addressed the following domains: (1) Decision making for first biopsy (who decides on necessity of biopsy); (2) Collaboration (consultation of rheumatologists by nephrologists and vice versa, use of multidisciplinary conferences to discuss biopsy results and treatment); (3) Decision making for repeat biopsy (for re-assessing histopathology changes in cases of refractory disease, response to therapy, and/or relapse); and (4) Biopsy procedures (who performs biopsy, availability of dedicated renal pathologist, pre-and post-biopsy laboratory assessment, overnight-stay vs. same-day discharge, classification systems, and use of activity and chronicity indices). This survey and the data analysis plan for this experimental research reported in the manuscript was performed with the approval of the Institutional Review Board at Northwestern University.

Hematuria is defined as ≥ 5 RBCs per high power field. Protocol biopsy is defined as a routinely scheduled biopsy performed on defined sets of pSLE patients for the purpose of modifying immunosuppression therapy, rather than a biopsy performed in response to changes in clinical or laboratory monitoring of patients.

Statistical approach: Groups were compared with Fischer’s exact test for categorical variables, and student *t* test for continuous variables. Significance was set at *p* = 0.05.

## Results

There were 182 respondents to the survey. Of these, 39 % indicated that they were pediatric nephrologists and 51 % were pediatric rheumatologists. There were no dual boarded pediatric nephrologist/rheumatologists who responded to the survey. Response rates were approximately 44 % of all pediatric nephrologists and 37 % of all pediatric rheumatologists. Of respondents who disclosed their primary institution, at least 65 of the 92 pediatric rheumatology centers involved in CARRA (71 %) and 34 of the 51 pediatric nephrology centers (67 %) with membership in the MWPNC were represented. Some institutions were represented by more than one respondent in one or both sub-specialty. Respondents were evenly distributed across the range of years in practice, with about a quarter each practicing 0–5, 6–10, 11–20, or >20years in their specialty (Table 2). There was no difference in years in practice between pediatric nephrologists and rheumatologists.

**Table 2:** Provider and center characteristics are comparable between groups*

	Pediatric Rheumatologists	Pediatric Nephrologists
Respondents	111	71
‘How long have you been practicing your specialty?’		
<5 years	26 (23 %)	18 (26 %)
6-10 years	27 (24 %)	19 (27 %)
11-20 years	29 (26 %)	15 (21 %)
>20 years	29 (26 %)	18 (26 %)
‘How many new pSLE patients per year are seen at your institution?’		
<5	16 (15 %)	12 (18 %)
6-10	37 (34 %)	24 (36 %)
11-20	38 (35 %)	14 (21 %)
>20	19 (17 %)	16 (24 %)
'How many kidney biopsies on pSLE patients are performed each year at your institution?’		
<5	41 (38 %)	24 (35 %)
6-10	43 (40 %)	24 (35 %)
11-20	21 (19 %)	16 (24 %)
>20	3 (3 %)	4 (6 %)

*Numbers (and percentage) of each group of sub-specialist are subdivided by the number of years practicing their specialty, recall on the number of new patients seen at their center in a typical year, and recall on the typical number of kidney biopsies performed (initial and repeat) per year

Respondents were representative of pediatric centers with a broad range of pSLE populations, based on the number of cases presenting each year. Four percent of respondents reported that their institution performs biopsies on > 20 pediatric patients per year; 21.0 %, on 11–20 patients per year; 38.1 %, on 6–10 patients per year; and 36.9 %, on fewer than 5 patients per year.

### Decision making for first biopsy

Results from our survey show that 78 % of respondents intend to follow ACR adult guidelines for recommending kidney biopsy in their pSLE patients (Table 3), equally divided by pediatric subspecialty. However, when asked about specific scenarios, only 28.8 % actually followed the guidelines. Hematuria alone warranted biopsy according to 25.0 %; proteinuria alone (defined as >150 mg/24h or a spot urine protein/creatinine ratio > 0.2 mg/mg), according to 58.2 %. Two pediatric rheumatologists and 2 pediatric nephrologists (each at separate institutions) routinely recommend or perform kidney biopsy in any patient with a new diagnosis of pSLE, regardless of the clinical findings.

**Table 3:** Tendency to agree on when to deviate from ACR guidelines for first biopsy in pSLE

	Pediatric Rheumatologists	Pediatric Nephrologists
Respondents	108	69
“Do you follow ACR guidelines for deciding to obtain a first kidney biopsy in a SLE patient” *		
Yes	83 (77 %)	56 (81 %)
No	25 (23 %)	13 (19 %)
Affirmative responses, sub-divided by years in practice		
<5	17 (68 %)	15 (83 %)
6-10	21 (78 %)	14 (78 %)
11-20	26 (90 %)	14 (100 %)
>20	19 (70 %)	12 (67 %)
unspecified		1 (100 %)
“Do you deviate from the ACR guidelines in your decision to obtain a first kidney biopsy? If so under which circumstances?”		
Affirmative responses for hematuria only	37 (34 %)	7 (10 %)
Affirmative responses for proteinuria only (>150mg/day and/or UPC > 0.2mg/mg)	64 (59 %)	40 (58 %)

*American College of Rheumatology (ACR) guidelines (see Table 1)were provided in the survey

**Table 4:** Variability between verses among pediatric rheumatologists and pediatric nephrologists in decision to perform repeat biopsy

	Pediatric Rheumatologists	Pediatric Nephrologists
Respondents*	109	68
“When do you repeat a kidney biopsy in a patient with proliferative SLE nephritis?		
“After the initial induction period, regardless of response to treatment”	7 (6 %)	11 (16 %)
“After the initial induction period, only if there is no response to treatment”	44 (40 %)	31 (46 %)
“After the initial induction period, if there is only partial response to treatment”	26 (24 %)	19 (28 %)
“After remission and before withdrawal of all immunosuppression”	6 (6 %)	3 (4 %)
“After lupus flare with worsening in urine sediment, proteinuria, or kidney function”	75 (69 %)	47 (69 %)
“I do not routinely perform a repeat biopsy in lupus nephritis patients”	35 (32 %)	17 (25 %)
“When do you repeat a kidney biopsy in a patient with membranous SLE nephritis?		
“After the initial induction period, regardless of response to treatment”	4 (4 %)	2 (3 %)
“After the initial induction period, only if there is no response to treatment”	33 (31 %)	24 (36 %)
“After the initial induction period, if there is only partial response to treatment”	14 (13 %)	12 (18 %)
“After remission and before withdrawal of all immunosuppression”	6 (6 %)	2 (3 %)
“After lupus flare with worsening in urine sediment, proteinuria, or kidney function”	71 (66 %)	48 (72 %)
“I do not routinely perform a repeat biopsy in lupus nephritis patients”	37 (35 %)	17 (25 %)

*Number and percentage of respondents indicating affirmatively that they would recommend repeat kidney biopsy in patients with pSLE, either for proliferative or membranous LN

Most respondents (66.5 %) have their patients’ biopsies read by a dedicated renal pathologist at their primary institution, although 21.2 % use a local general pathologist. The remaining 12.3 % have their biopsies sent out to a renal pathologist at another institution. Pathologists utilize the WHO grading system (33.2 %), the ISN/RPS grading system (22.4 %), or both (33.0 %). A total of 9.5 % of respondents were unsure of which grading system their pathologists were using (11.6 % of nephrologists vs. 8.2 % of rheumatologists). For diffuse proliferative GN, the activity and chronicity indices were provided routinely to 80.9 % of respondents, whereas 2.3 % received only the activity index, 1.1 % received only the chronicity index, and 15.2 % worked with pathologists who did not provide either.

### Collaboration

More than 90 % of pediatric rheumatologists and nephrologists refer their patients with pSLE to the other provider after a diagnosis of nephritis is made. There are very few pediatric nephrologists who do not have access to a pediatric rheumatologist at their primary institution (3.9 %, *n* = 3), and 2 of those 3 have an internal medicine rheumatologist at their institution who care for children. Similarly, only 3.6 % of pediatric rheumatologists (*n* = 4) do not have access to a pediatric nephrologist at their primary institution. The decision to perform a kidney biopsy in a patient with pSLE is made by 62.5 % of respondents only after discussions are held between nephrology and rheumatology. Only 29.6 % of respondents stated that the decision was made by the pediatric nephrologists alone; and only 7.8 %, by the pediatric rheumatologist alone.

Pediatric rheumatologists report viewing the tissue slides together with their pediatric nephrologist and pathologist 49.1 % of the time and without a pathologist 2.7 % of the time. Pediatric rheumatologists report reviewing tissue section slides 14.6 % of the time with the pathologist separately from nephrology, and 33.4 % of pediatric rheumatologists review only the biopsy report.

### Decision making for repeat biopsy

There were no significant differences in the proportion of pediatric rheumatologists or nephrologists that would recommend/perform repeat renal biopsies under specific instances (Table 4). For proliferative LN (class III or IV), there were 10 % (*n* = 18) who obtain true “protocol” biopsies in order to guide the dosing of induction therapy, and 5 % who re-biopsy patients in remission in order to guide withdrawal of immunosuppression. However, 41.7 % would obtain repeat kidney biopsy after induction therapy in patients with no response and 25 % would in patients with only partial response. For patients in renal remission from a proliferative nephritis who develop a renal flare, 69 % would obtain another biopsy to guide therapy. Only 2.7 % (*n* = 5) obtain repeat biopsy for non-renal lupus flares.

There were 29 % of respondents who stated that they do not routinely repeat kidney biopsy after an initial pathologic diagnosis of proliferative LN. Of these 17 were nephrologists and 35 were rheumatologists. Of the nephrologists who do not re-biopsy, most stated that they made biopsy decisions alone without consultation of their rheumatology colleagues. Of the rheumatologists who do not re-biopsy, only 2 stated that they made biopsy decisions alone, and most did so in consultation with their local pediatric nephrologists. The pediatric rheumatologists who do not recommend routine repeat biopsy tended to have been practicing longer, whereas the pediatric nephrologists who do not perform routine repeat kidney biopsy tended to have been practicing for < 10 years. All 16 pediatric nephrologists practicing their specialty >20 years stated that they will routinely re-biopsy patients with proliferative LN for specific indications.

Repeat biopsy after initial diagnosis of membranous LN (class V) differed very little from the practice following initial diagnosis of proliferative LN (Table 3). Only 32.0 % would obtain repeat kidney biopsy after induction therapy in patients with no response and 14.7 % would in patients with only partial response. There were no significant differences to the responses received from nephrologists compared to rheumatologists.

### Biopsy procedure

The majority (71.3 %) of respondents reported that at their institutions, renal biopsies on pSLE patients with nephritis were primarily performed by pediatric nephrologists. There were 19.9 % of respondents from institutions where interventional radiology primarily performed the biopsies, and respondents from the remaining 8.8 % of institutions had both pediatric nephrologists and radiologists performing proportionate numbers of biopsies.

Pediatric nephrologists were also questioned about their preferences regarding the biopsy procedure itself. When asked about pre-procedural laboratory testing, complete blood count (CBC) is usually performed by 96 % of nephrologists, partial thromboplastin time (PTT) by 94 %, prothrombin time (PT) by 90 %, and INR by 76 %. Less common laboratory testing included: type and screen (36 %), platelet function testing (15 %), lupus anticoagulant (12.6 %), serum chemistries (2.8 %), urine beta-HCG (2.8 %), urinalysis (1.4 %), and bleeding time (1.4 %). Significant anemia (<7 g/dL) is thought to be a contraindication to kidney biopsy by 35.4 % of nephrologists, whereas 5.4 % delay biopsy for Hgb < 8 g/dL and 1.4 % delay biopsy for Hgb < 9 g/dL until anemia can be corrected. For significant thrombocytopenia (<50x10^3/mcL) 58 % of nephrologists will defer biopsy. A few specifically commented that they would transfuse PRBC, platelets, or FFP in order to perform kidney biopsy without delay. Other contraindications to renal biopsy included active infection (58 %), uncontrolled hypertension (59 %), and solitary kidney (17 %).

Non-steroidal anti-inflammatories (NSAIDs), including aspirin, are routinely held by 85 % of pediatric nephrologists prior to biopsy. Most recommended holding these drugs for 3–7 days prior to kidney biopsy (54.4 %), whereas 1.5 % held for 1 day, 11.8 % held for 2 days, 11.8 % held 8–14 days, and 5.9 % held >14 days. NSAID use shortly before kidney biopsy is considered an absolute contra-indication by 27 % of nephrologists. However, 14.7 % of pediatric nephrologists would proceed with biopsy despite NSAID use even for routine non-emergent kidney biopsies. Some pediatric nephrologists consider recent NSAID use a higher risk procedure warranting referral to interventional radiology. Others start immunosuppressive therapy and delay biopsy because of the NSAID use.

**Fig. 1:**
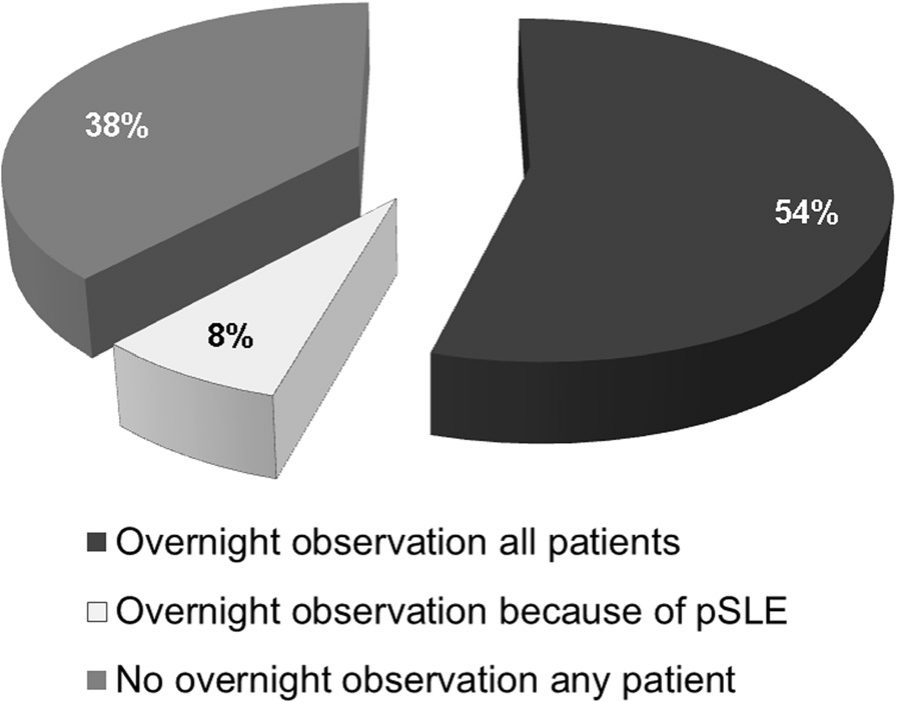
A significant proportion of respondents manage pSLE patients differently post-biopsy. Pediatric nephrologists were asked when they typically discharged patients after kidney biopsy, in the absence of complications. Most “keep all patients overnight for observation, regardless of whether they have SLE,” as opposed to discharging them home same day. However, 8 % “keep SLE patients after kidney biopsy for overnight observation solely because of possible increased risks associated with SLE.”

Of 68 pediatric nephrologists, 54.4 % routinely observe patients overnight after kidney biopsy, whereas 38.2 % routinely send patients without complications home on the same day. Some nephrologists have particular concerns about pSLE patients after biopsy, as suggested by the fact that 7.4 % routinely observed their pSLE patients, but not patients with non-SLE kidney disease, overnight before discharge (Fig. 1).

## Discussion

Results of this survey of North American pediatric subspecialists clearly indicate that rheumatologists and nephrologists do frequently work together in the care of patients with pSLE and nephritis. Most pediatric nephrologists and rheumatologists routinely review biopsy results together as well. However there is a wide variation in approaches to performing and interpreting biopsies in pSLE among both sub-specialties.

### Decision making for first biopsy

Pediatric rheumatologists and nephrologists appear to value guidelines such as those published by the ACR, based on the high proportion who stated that they use them. However, the number of respondents who deviated based on pediatric definitions of proteinuria suggests that pediatric guidelines might be useful. The numbers of respondents who deviated were comparable between specialties. The differences in opinion on when to biopsy relate to number of years in practice in a more complex manner. There were approximately equal proportions of both pediatric nephrologists and pediatric rheumatologists in each of the 4 groups (0–5 years, 6–10 years, 11–20 years, and > 20 years) (*p*-value = 0.114). The only significant correlation between years in practice and practice patterns was between the proportion of individuals who had been practicing 11–20 years and adherence to ACR guidelines (93 % compared to 70-76 % in the other groups, *p*-value = 0.029).

### Collaboration

The results of the survey show that it is uncommon for pediatric nephrologists to manage LN without input from pediatric rheumatologists. Of pediatric nephrologists responding, 90 % refer even their patients with renal-limited pSLE to rheumatology before or after a diagnosis of LN is made. If extra-renal manifestations are also present, 97 % refer. Moreover, rheumatologists are consulted by over 2/3rds of pediatric nephrologists in making the decision to perform kidney biopsy, and over half view the biopsy slides along with their pediatric rheumatology colleagues.

There are also very few pediatric rheumatologists (<5 %) who manage nephritis in pSLE without routinely seeking the input of pediatric nephrologyOf these 6 rheumatologists, 3 make the decision to biopsy without other consultation, but the other 3 seek guidance from their nephrologist when deciding to order a kidney biopsy, and 5 have their nephrologist perform the biopsy once the decision is made. In addition, 5 of the 6 review renal biopsy results with their local pediatric nephrologist.

### Decision making for repeat biopsy

There is clearly no consensus regarding the indications for repeat kidney biopsy in the management of proliferative or membranous LN. Just under one third of respondents do not routinely recommend repeat biopsy in any instance. Alternatively, 1.7 % routinely recommend repeat biopsies for any lupus flare, regardless of kidney involvement. The most common practice is to repeat kidney biopsy in patients who have an exacerbation of their lupus which results in worsening of the urinary sediment (2/3rds of respondents). The second most common is to repeat biopsy at some time point during induction therapy in non-responders (32 % for membranous LN and 42 % for proliferative LN). Only a few programs use “protocol” biopsies to guide duration of induction or maintenance therapies in patients regardless of response. Interestingly, there were no significant differences in the proportion of rheumatologists or nephrologists that engaged in the practice of recommending/performing routine “protocol” repeat biopsy. This suggests that in general pediatric rheumatologists and nephrologists are similar in their approach to repeat biopsy, and that differences that exist are more common between individual physicians than between sub-specialties.

### Biopsy procedures

Despite the ongoing role of pediatric nephrologists in medical management of nephritis in pSLE, there might be a shift in who is actually performing the biopsies. Many nephrologists appear to consider pSLE to be a risk factor for complications after kidney biopsy, and 12 (17 %) are primarily referring their pSLE patients to interventional radiology to perform the biopsy. It was not asked whether these nephrologists perform kidney biopsies routinely on their other patients. Of rheumatologists who completed the survey, referral rates to nephrology and interventional radiology are comparable. Therefore only 70 % of pediatric nephrologists are choosing to perform biopsies of patients with pSLE and only 70 % of pediatric rheumatologists are having their local pediatric nephrologist performing the biopsies.

Finally, there is great variability in the perceived safety of kidney biopsies in pSLE. Most of this variability is likely to reflect differences in perceived safety of kidney biopsy in general. There are 38 % of pediatric nephrologists who discharge their pSLE patients the same day as the procedure, similar to their practice with non-SLE patients. Conversely, 54 % observe both their pSLE and non-SLE patients overnight. However, 8 % of pediatric nephrologists do send most patients home on the day of biopsy, but specifically observe pSLE patients overnight, due to perceived risk of complications. Identifying sources of perceived increased risk of kidney biopsy in children with lupus nephritis was beyond the scope of this study, but hypotheses can be drawn from nephrologist responses to survey questions on biopsy procedure. Possibilities include higher rates of anemia, thrombocytopenia, hypertension, NSAID use, or anticoagulant use in pSLE patients than in non-SLE patients, as well as a perceived need for performing biopsy more urgently and less electively when relative contraindications exist,

Limitations of our study include the possibility of selection bias, recall bias, or reporting bias. Results of survey-based studies can be inherently subject to significant bias, and the strategies for survey distribution as well as the response rates should always be taken into account when interpreting survey data. Questions were written by pediatric nephrologists and rheumatologists together in order to minimize leading questions or error due to differences in terminology. By surveying specialists participating in either CARRA or MWPNC, we may have biased the results towards particular types of pediatric centers. We chose to survey providers as opposed to centers, as differences in practice patterns amongst providers in the same center are often encountered. There were no restrictions on how many providers could respond to the survey from any one center, which could allow for bias. However, the large degree of voluntary reporting of the respondents’ institution allowed us to compare practice patterns within centers with the highest degree of survey participation. The degree of collaboration between sub-specialties may not be applicable to primary care pediatric centers or centers outside of the United States or Canada. However, the strengths of the survey include the >40 % response rate, the large number of institutions represented, and the high proportion of completed surveys without missing responses. Aside from reports of transplant kidney biopsy, this study represents one of the only surveys to assess approach to kidney biopsy for a particular kidney disease, lupus nephritis, and the only survey to date that compares the approaches between pediatric rheumatologists and nephrologists.

## Conclusions

The results of this survey support the need for developing a pSLE-specific approach to kidney biopsy. Agreement between pediatric nephrology and rheumatology should not be an impediment. We support the formation of a collaborative group consisting of both pediatric nephrologists and pediatric rheumatologists to find ways to improve the diagnosis and treatment of LN and promote more collaborative research (PNR-CG, the Pediatric Nephrology and Rheumatology Collaborative Group). Studies on the effect of different strategies for performing repeat biopsy on the outcomes of proliferative and membranous nephritis in pSLE are warranted. Variability around practice remains significant and it is becoming clear in medicine that standardization can greatly improve outcomes.

## Additional files

Additional file 1: ScreenCaptures of the SurveyMonkey querying Pediatric Rheumatologists ᅟ Additional file 2: ScreenCaptures of the SurveyMonkey querying Pediatric Nephrologists ᅟ

## Abbreviations

ACR: American College of Rheumatology
AI: Activity index
CARRA: Childhood Arthritis and Rheumatology Research Alliance
CI: Chronicity index
CKD: Chronic kidney disease
EULAR: Joint European League Against Rheumatism
ERA-EDTA: European Renal Association-European Dialysis and Transplant Association
GN: glomerulonephritis
ISN: International Society of Nephrology
LN: Lupus nephritis
MWPNC: Midwest Pediatric Nephrology Consortium
NSAID: Non-steroidal anti-inflammatory
pSLE: Pediatric systemic lupus erythematosus
RPS: Renal Pathology Society
WHO: World Health Organization (WHO)
