# Supplementary material for: Practice patterns and approach to kidney biopsy in lupus: a collaboration of the Midwest pediatric nephrology consortium and the childhood arthritis and rheumatology research alliance
Source: Pediatr Rheumatol Online J. 2015 Jun 19;13:26. doi: 10.1186/s12969-015-0024-x (PMC4474548; doi:10.1186/s12969-015-0024-x)
Supplement: Additional file 1: ScreenCaptures of the SurveyMonkey querying Pediatric Rheumatologists — ᅟ [file 12969_2015_24_MOESM1_ESM.pdf]

[SURVEY PREVIEW MODE] Version 2 SURVEY OF PRACTICE PATTERNS FOR KIDNEY BIOPSY OF SLE PATIENTS - Windows Internet Explor...

HIS_LINK_FOR_COLLECTION&sm=ZwdLp7X%2f4yk3HkJhnaEKPICOp2ZK2qkj2XDbQXr%2fTMQ%3d SurveyMonkey, Inc [US]

Convert Select

Version 2 SURVEY OF PRACTICE PATTERNS FOR KIDNEY BIOPSY OF SLE PATIENTS

5%

The following survey is a collaboration between Pediatric Nephrologists and Rheumatologists. The purpose of this survey is to assess practices and practice variations regarding kidney biopsy for patients with lupus nephritis. This survey will be answered by both nephrologists and rheumatologists, although a few questions will be specific for your indicated specialty. For the purposes of this survey, pediatric patients are defined as being ≤ 21 years of age. If you practice in more than one hospital, please respond regarding your primary institution.

Next

Powered by SurveyMonkey
Check out our sample surveys and create your own now!

75%

[SURVEY PREVIEW MODE] Version 2 SURVEY OF PRACTICE PATTERNS FOR KIDNEY BIOPSY OF SLE PATIENTS - Windows Internet Explor...

https://www.surveymonkey.com/s.aspx?PREVIEW_MODE=DO_NOT_USE_THIS_LINK_FOR_COLLECTION&sm=ZwdLp7X%2f4 SurveyMonkey, Inc [US]

Convert Select

Version 2 SURVEY OF PRACTICE PATTERNS FOR KIDNEY BIOPSY OF SLE PATIENTS

10%

1. What is your specialty?

- Pediatric Nephrology
- Pediatric Rheumatology

Prev Next

Powered by SurveyMonkey
Check out our sample surveys and create your own now!

75%

[SURVEY PREVIEW MODE] Version 2 SURVEY OF PRACTICE PATTERNS FOR KIDNEY BIOPSY OF SLE PATIENTS - Windows Internet Explor...

https://www.surveymonkey.com/s.aspx?PREVIEW_MODE=DO_NOT_USE_THIS_LINK_FOR_COLLECTION&sm=ZwdLp7X%2f4 SurveyMonkey, Inc [US]

Convert Select

Version 2 SURVEY OF PRACTICE PATTERNS FOR KIDNEY BIOPSY OF SLE PATIENTS

67%

2. How long have you been practicing your specialty?

- 0-5 years
- 6-10 years
- 11-20 years
- > 20 years

3. Who performs the majority (>50%) of kidney biopsies of SLE patients at your institution?

- Pediatric Nephrology
- Interventional Radiology
- Both

4. Where is your institution located?

- USA/Canada
- Europe
- Mexico/Central/South America
- Asia

Prev Next

Powered by SurveyMonkey
Check out our sample surveys and create your own now!

75%

[SURVEY PREVIEW MODE] Version 2 SURVEY OF PRACTICE PATTERNS FOR KIDNEY BIOPSY OF SLE PATIENTS - Windows Internet Explor...

https://www.surveymonkey.com/s.aspx?PREVIEW_MODE=DO_NOT_USE_THIS_LINK_FOR_COLLECTION&sm=ZwdLp7X%2f4 SurveyMonkey, Inc [US]

Convert Select

Version 2 SURVEY OF PRACTICE PATTERNS FOR KIDNEY BIOPSY OF SLE PATIENTS

Copy of page:

71%

5. Is there a pediatric nephrologist at your institution?

- Yes
- No

6. Approximately how many new pediatric patients with SLE (with and without nephritis) are seen each year at your institution?

- 0-5
- 6-10
- 11-20
- More than 20

7. Approximately how many biopsies on pediatric patients with SLE are performed each year at your institution?

- 0-5
- 6-10
- 11-20
- More than 20

8. At your institution, how are the majority (> 50% of the time) of decisions regarding whether to perform a kidney biopsy for a patient with SLE made?

- The decision is made primarily by Pediatric Nephrology
- The decision is made primarily by Pediatric Rheumatology
- The decision is made only after discussion between Pediatric Nephrology and Rheumatology

9. Do you always refer patients with lupus nephritis (LN) to Nephrology?

- Yes
- No

Prev Next

66%

[SURVEY PREVIEW MODE] Version 2 SURVEY OF PRACTICE PATTERNS FOR KIDNEY BIOPSY OF SLE PATIENTS - Windows Internet Explor...
https://www.surveymonkey.com/s.aspx?PREVIEW_MODE=DO_NOT_USE_THIS_LINK_FOR_COLLECTION&sm=ZwdLp7X%2f4 SurveyMonkey, Inc [US]
Convert Select

Version 2 SURVEY OF PRACTICE PATTERNS FOR KIDNEY BIOPSY OF SLE PATIENTS

Copy of page:

76%

10. Do you obtain a kidney biopsy for all patients with SLE at initial presentation, even with normal urinalysis and kidney function?

- ○ Yes
- ○ No

Prev Next

Powered by SurveyMonkey
Check out our sample surveys and create your own now!

75%

[SURVEY PREVIEW MODE] Version 2 SURVEY OF PRACTICE PATTERNS FOR KIDNEY BIOPSY OF SLE PATIENTS - Windows Internet Explor...

https://www.surveymonkey.com/s.aspx?PREVIEW_MODE=DO_NOT_USE_THIS_LINK_FOR_COLLECTION&sm=ZwdLp7X%2f4yk Identified by VeriSign

Convert Select

Version 2 SURVEY OF PRACTICE PATTERNS FOR KIDNEY BIOPSY OF SLE PATIENTS

Copy of page:

81%

11. Do you follow the ACR guidelines for deciding to obtain a first kidney biopsy in an SLE patient as listed?

American College of Rheumatology (ACR) Biopsy Guidelines:
*** Increasing serum creatinine without compelling alternative causes (such as sepsis, hypovolemia, or medication)**

*** Confirmed proteinuria of ≥ 1.0 gm / 24 hours (either 24-hour urine specimens or spot protein/creatinine ratios are acceptable)**

*** Combination of 1) proteinuria ≥ 0.5 gm / 24-hrs plus 2) hematuria (defined as ≥5 RBCs per hpf), confirmed in at least 2 tests done within a short period of time, in the absence of alternative causes**

*** Combination of 1) proteinuria ≥ 0.5 gm / 24-hrs plus 2) cellular casts, confirmed in at least 2 tests done within a short period of time, in the absence of alternative causes**

- ○ Yes
- ○ No

12. Do you deviate from the ACR guidelines in your decision to obtain a first kidney biopsy in an SLE patient? If so, under what circumstances would you obtain a first kidney biopsy in an SLE patient? (check all that apply):

- ☐ Hematuria only (> 5 rbc/hpf).
- ☐ Proteinuria only (>150 mg/24 hr and/or urine protein/creatinine > 0.2 mg/mg)
- ☐ I do not deviate from ACR guidelines

13. For pathology readings of SLE kidney biopsies, does your institution use:

- ○ A dedicated renal pathologist
- ○ General pathologist
- ○ Biopsies are sent out to a general pathologist at another institution
- ○ Biopsies are sent out to a renal pathologist at another institution

Prev Next

66%

Version 2 SURVEY OF PRACTICE PATTERNS FOR KIDNEY BIOPSY OF SLE PATIENTS

Copy of page:

88%

14. For Rheumatologists answering this survey, do you review results of kidney biopsies of SLE patients:

- We do not review biopsy results (reports or slides)
- We review written biopsy reports only
- We review tissue slides with Pathology only
- We review tissue slides with Pathology and Nephrology
- We review tissue slides with Nephrology only

15. What grading system of SLE nephritis does your institution use?

- WHO
- ISN/RPS
- Both
- I don't know

16. Do your kidney biopsy reports contain:

- Activity index only
- Chronicity index only
- Both
- Neither

Prev Next

Powered by SurveyMonkey
Check out our sample surveys and create your own now!

[SURVEY PREVIEW MODE] Version 2 SURVEY OF PRACTICE PATTERNS FOR KIDNEY BIOPSY OF SLE PATIENTS - Windows Internet Explor...
https://www.surveymonkey.com/s.aspx?PREVIEW_MODE=DO_NOT_USE_THIS_LINK_FOR_COLLECTION&sm=ZwdLp7X%2f4 SurveyMonkey, Inc [US]
Convert Select

Version 2 SURVEY OF PRACTICE PATTERNS FOR KIDNEY BIOPSY OF SLE PATIENTS

Copy of page:

90%

17. When do you recommend / arrange for repeat kidney biopsy in a patient with proliferative SLE nephritis? (check all that apply)

- [ ] I perform protocol biopsies at regularly defined intervals regardless of laboratory findings (e.g. at 6 months, 1 year, and or two years, etc.)
- [ ] After the initial induction period, regardless of response to treatment
- [ ] After the initial induction period, only if there is no response to treatment
- [ ] After the initial induction period, if there is only partial response to treatment
- [ ] After a lupus flare without change in kidney indices (urinalysis and creatinine)
- [ ] After lupus flare with worsening in urine sediment, proteinuria or kidney function
- [ ] After remission and before withdrawal of all immunosuppression
- [ ] I do not routinely perform a repeat biopsy in lupus nephritis patients

Prev Next

Powered by SurveyMonkey
Check out our sample surveys and create your own now!

75%

[SURVEY PREVIEW MODE] Version 2 SURVEY OF PRACTICE PATTERNS FOR KIDNEY BIOPSY OF SLE PATIENTS - Windows Internet Explor...

https://www.surveymonkey.com/s.aspx?PREVIEW_MODE=DO_NOT_USE_THIS_LINK_FOR_COLLECTION&sm=ZwdLp7X%2f4 SurveyMonkey, Inc [US]

Convert Select

Version 2 SURVEY OF PRACTICE PATTERNS FOR KIDNEY BIOPSY OF SLE PATIENTS

Copy of page:

95%

18. When do you repeat a kidney biopsy in a patient with membranous SLE nephritis? (check all that apply)

- ☐ I perform protocol biopsies at regularly defined intervals regardless of laboratory findings (e.g. at 6 months, 1 year, and or two years, etc.)
- ☐ After 6-12 months, regardless of response to treatment
- ☐ After 6-12 months, only if there is no response to treatment
- ☐ After 6-12 months, if there is only partial response to treatment
- ☐ After a lupus flare without change in kidney indices (urinalysis and creatinine)
- ☐ After lupus flare with worsening in urine sediment, proteinuria or kidney function
- ☐ After remission and before withdrawal of all immunosuppression
- ☐ I do not routinely recommend repeat biopsy in lupus nephritis patients

Prev Next

Powered by SurveyMonkey
Check out our sample surveys and create your own now!

75%

[SURVEY PREVIEW MODE] Version 2 SURVEY OF PRACTICE PATTERNS FOR KIDNEY BIOPSY OF SLE PATIENTS - Windows Internet Explor...

https://www.surveymonkey.com/s.aspx?PREVIEW_MODE=DO_NOT_USE_THIS_LINK_FOR_COLLECTION&sm=ZwdLp7X%2f4 SurveyMonkey, Inc [US]

Convert Select

Version 2 SURVEY OF PRACTICE PATTERNS FOR KIDNEY BIOPSY OF SLE PATIENTS

Copy of page:

100%

19. What is the name of your primary institution?

20. Are there any other special considerations regarding kidney biopsy in pediatric SLE patients that are not mentioned in this survey?

21. Do you have any other comments?

Prev Done

Powered by SurveyMonkey
Check out our sample surveys and create your own now!

75%
